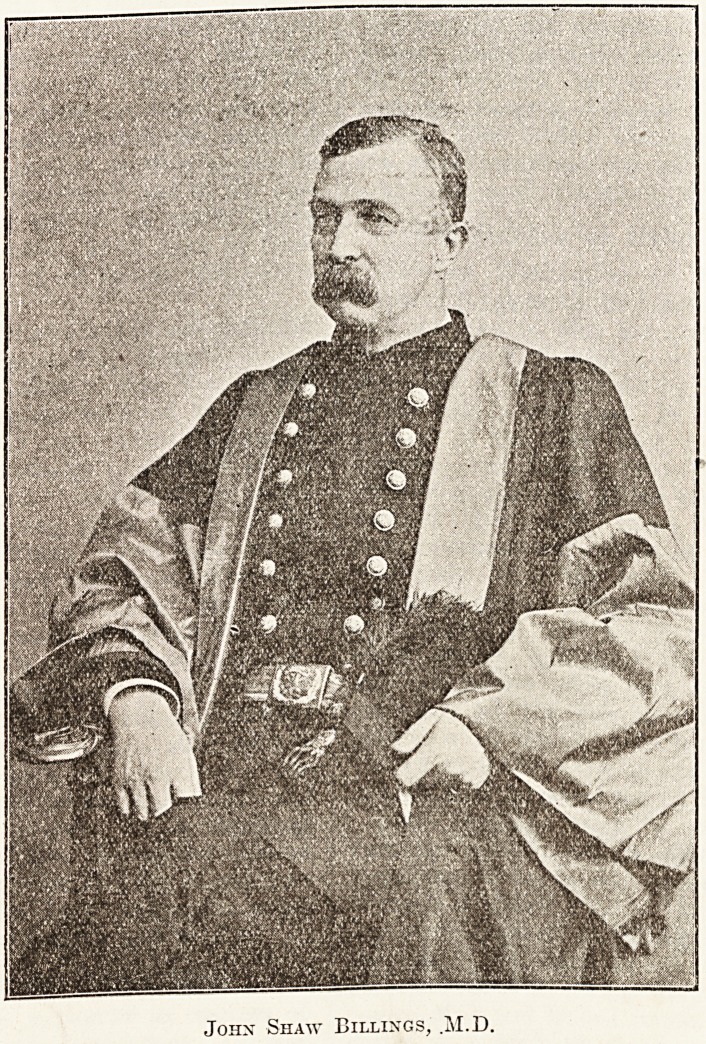# A Great Hospital Authority: Colonel John Shaw Billings

**Published:** 1913-03-22

**Authors:** 


### A GREAT HOSPITAL AUTHORITY.

### Colonel John Shaw Billings, M.D., of the United States Army.

It is with keen personal regret that we have to report the death of Colonel John Shaw Billings, M.D., in New York on the 11th inst. in his seventy-fifth year. He was certainly one of the kindest, most competent, lovable, unselfish, and bravest men of his day and generation. His powers of work were enormous, and no one who had the opportunity, as the present writer had, of spending several days with him when engaged on it could fail to be impressed with his extraordinary organising powers, his self-discipline, and genius for method. Such association was an inspiration and encouragement to the best minds and most successful workers of the day.

John Billings, as he was familiarly designated by all those who knew him best, was called upon to suffer and endure face to face the effects of disease and peril to life to an extent altogether beyond the normal lot of average people. It is an extraordinary thing how many of the ablest men, whose lives, humanly speaking, seem of paramount importance to the community, are called upon to face ill-health and to suffer more perils than those which fall to the lot of average humanity. Nature is rarely prodigal of all her gifts, or careful of her best lives. John Billings was certainly one of these. Attacked by cancer many years ago, he twice underwent an operation without the knowledge of his wife, to whom he was devotedly attached, and came back again to his home, took up his ordinary work in so ordinary a manner that Mrs. Billings, one of the most delightful of women, never had an idea of the suffering and perils which he had gone through during what he denominated a short vacation. Later on, when he found himself attacked by a grievous internal trouble, for which orthodox treatment failed, he determined to place himself again in the hands of the surgeons, and was one of the first to undergo an internal operation of so dangerous and complicated a character that at that time few surgeons would undertake it. Each time he survived the operation, having a wonderful constitution, and returned to his work, which he was able to pursue with uninterrupted success until the day of his death. The sufferings and trials which John Billings endured, having regard to the value of his life and health to the generation which he served, belong to those mysteries which pass human comprehension. No doubt in God's mercy there was purpose in it all, for everything to a nature like his tended to make him a great character, full of the highest and noblest influence on every life which had the good fortune to come in contact with his own. We are conscious that a knowledge of what he had to suffer and of the spirit in which he bore it all has made it easier to some of his contemporaries to face peril or temptation, and if ever the temptation to shirk has come, his example has put fibre and manliness into many of us, and made us feel that whatever the cost, seeing what he did and how nobly he bore all his trials, we at least as his friends could not do less than our best always, though the heavens fall. The loss of such a life to his family and friends is irreparable, but it is a glorious and grateful fact to remember that John Billings was permitted to continue in harness to the end, though he was nearly seventy-five years of age. The loss of his wife last autumn was keenly felt. Writing afterwards he records that "the end came in a peaceful, quiet sleep. She saw all the children within two weeks before her death and was happy. I hope that in my finale

John Shaw Billings, M.D.

I may be as patient and cheerful as she was." He adds: "Probably I shall be retired from the Library work in 1913; I shall be seventy-five years old in April of that year, and it is quite useless to try to make any plans now, nor do I feel like doing so." His last written words to the writer were, referring to the card sent him last Christmas which appealed to him by its advice, "If troubles arise promptly sit on 'em," were "I shall try to act upon it," as he certainly invariably did.

We have written this personal note on one of the greatest and most useful workers the world has lately seen, because his memory and example must ever be fruitful for good to those of us who remain and to those who come after. It would be refreshing and helpful to many a hospital worker, no matter in what part of the world he may be working, to read or read again the late Dr. Billings' paper entitled "Hospital Construction and Organisation," being one of five essays contributed for the use of the Johns Hopkins Hospital, Baltimore, published by William Wood and Co., New York, and Sampson Low, Marston and Co., London, in 1875. In that year and the next the late Dr. Billings spent many months in visiting and inspecting the principal hospitals, universities, and medical schools in this country and throughout Europe. He came, nearly forty years ago, on his first visit with a schedule of plans embodying his ideas as to what a modern hospital ought to be, and it was the writer's privilege to co-operate with him to the extent of writing upon those plans the different hospitals and places where Dr. Billings would find some of these same ideas embodied in whole or in part in existing buildings. One of the remarkable sides of Dr. Billings was his power of getting at the root base of each language, so that he learnt how to extract the information he wanted from books and documents in almost any tongue, although he might be quite unable to speak the language.

### Dr. Billings' Idea of a Hospital.

It is well for historical reasons to point out that in 1875 Dr. Billings' idea of a hospital embraced many developments, then mostly novel, which forty years' experience have caused to be at present accepted as essentials to the efficient construction and administration of an up-to date modern hospital. He maintained that in addition to the succour of the sick and the education of practitioners and nurses, a properly organised clinical hospital, to be efficient, must promote discoveries in the science and art of medicine and make them known for the general good. In 1875, especially in the United States, though original investigators wished to pursue the path of knowledge, they generally lacked both the means and the time to do so. It was for this reason that his scheme for the Johns Hopkins Hospital, at Baltimore, included the appointment of professors of the highest eminence to Chairs in connection with its school, so that the profession and people of America and the world at large might look with confidence to it for the scientific observation and information so much needed then and now to increase our power of healing or comforting the sick. It is notable that at that period, when the training of nurses was only in its infancy, and enforced at very few hospitals, that he should have insisted upon the importance of establishing a training school for nurses in connection with the Baltimore Hospital, and that the nurses themselves should be housed in a separate building which should be near or on the hospital grounds. He was one of the first to insist upon the importance of teaching nurses in the course of their training how to prepare and serve food properly, and to carry this out so systematically as to ensure the instruction of the nurses in cooking in the diet kitchen attached to each ward. He was far-seeing enough to dwell upon the importance of providing accommodation of a special nature for pay and private patients of several grades as a part of the ordinary work and scheme of a great general hospital. The higher-class pay patients to be accommodated in suites of rooms entirely separated from the wards but near the administration building, the other pay patients of a lower grade to be provided for in connection with the wards. He properly attached the greatest importance to a system of records at each hospital —financial, historical, and professional—which must be made as complete as possible. He was one of the first to secure that the out-patient department should be connected with the buildings designed for the instruction of students but entirely separate from the main administration buildings. He never lost sight of the importance of insisting that the best interests of the patients must be the first consideration in every medical cure-house, and that the greater part of the clinical instruction should be given to students in the wards and in the out-patient department, and not in an amphitheatre attached to the ward blocks, as is done in most Continental hospitals, with, of course, the exception of the operation unit.

He emphasised the fact which it is most desirable to enforce in view of impending changes in our hospital system in this country, that it is seldom justifiable to bring a patient confined to his bed into the amphitheatre for any clinical purpose at all, and the physician-in-charge or medical superintendent of every hospital should have the power to prevent the taking of any given patient to the clinical amphitheatre. These views were enunciated in 1875, and had they then been universally adopted throughout the hospitals of the world who can estimate the amount of human suffering and loss of life in hospitals which would have been prevented? Another important point, mainly economical, but also of importance administratively, was his insistence that it is desirable, although not absolutely necessary, to have two pharmacies, one in connection with the out-patient department and one for the hospital proper. In a teaching hospital the existence of two pharmacies enables more places to be found for the training of students selected from those who have undergone some training already from the hospital apothecary.

### His Publications and Early Life.

Dr. John Billings was a pioneer in matters of isolation, of ventilation, and of epidemics. He was the author of many books and more pamphlets of great practical value. One of the most valuable is that on "The Principles of Ventilation and Heating," which should find a permanent place in the library of all who desire to keep continuously in touch with everything that is of the greatest importance to the maintenance and construction of hospitals. We have not space, nor would it fulfil any useful purpose, to enumerate the many hospital buildings and hygienic developments and systems in the United States in the construction or perfecting of which Dr. Billings had a part. Dr. John Billings had other great gifts, apart altogether from his hospital work. Graduating in Arts at Miami University in 1857, he completed his medical course in 1860 at the Medical College of Ohio, Cincinnati. The following year he was appointed an acting assistant surgeon when the war broke out between the North and South. He was medical inspector of the army of the Potomac; at the end of the war and up to 1875 he was attached to the Surgeon-General's Office in Washington, where he took charge of the library. This led to his being made curator of the Army Medical Museum and Library in 1863, which resulted in his making it probably the most complete and finest collection of medical works in the world. He designed and completed the first series of the Index Catalogue of this Library, of which there are sixteen volumes, and it was only the other day that we received Volume XVII. of the second series. This Index Catalogue is without doubt the most complete and exhaustive subject-bibliography of medical works in existence.

### The Museum of Hygiene he Founded.

Leaving Washington in 1891 he became Professor of Hygiene in the University of Pennsylvania, and we owe to him the magnificent Museum of Hygiene in Philadelphia, which was probably the first, and as we consider by far the best, museum of the kind in existence. In 1896 he was appointed director of the New York Public Library, Astor, Lennox, and Tilden Foundations, a post which he held at the time of his death. When he commenced this work these institutions required reorganisation and extension. He threw himself vigorously into the work, came frequently to Europe in search of rare books and missing volumes of importance, and by continuous industry and sheer ability, including the planning, erection, and completion of a new central building, the extension of the reference department and the provision of branch libraries, he has given the United States of America one of the most remarkable establishments of the kind anywhere.

John Billings was a great organiser, an untiring worker, and a born bibliophile, whose knowledge of books was so remarkable that Oliver Wendell Holmes said to the present writer one day when speaking of Billings: "Yes, I remember the man. He came to my house at Cambridge once and asked to see my library. He stood in the centre of it and looked round. Then he suddenly darted at a book on a certain shelf and began to examine it. It was the most valuable book in my collection. Replacing it he turned round, took another survey and made tracks for a second book, which was the second most valuable book in my possession. Why, sir, Dr. Billings is a bibliophile of such eminence that I regard him as a positive danger to the owner of a library if he is ever let loose in it alone." John Billings had probably more honorary degrees conferred upon him by the greater universities of the world than anyone of his generation. His memory will live throughout these seats of learning, and it should be an unfailing exemplar to all devoted hospital workers.

## Figures and Tables

**Figure f1:**